# 4-Hydroxynonenal in Redox Homeostasis of Gastrointestinal Mucosa: Implications for the Stomach in Health and Diseases

**DOI:** 10.3390/antiox7090118

**Published:** 2018-09-03

**Authors:** Andriy Cherkas, Neven Zarkovic

**Affiliations:** 1 Department of Internal Medicine #1, Danylo Halystkyi Lviv National Medical University, 79010 Lviv, Ukraine; cherkasandriy@yahoo.com; 2 Laboratory for Oxidative Stress (LabOS), Institute “Rudjer Boskovic”, HR-10000 Zagreb, Croatia

**Keywords:** 4-hydroxynonenal, lipid peroxidation, redox balance, oxidative stress, stomach, peptic ulcer, gastritis, *Helicobacter pylori*, gastric cancer, non-steroid anti-inflammatory drugs-induced gastropathy

## Abstract

Maintenance of integrity and function of the gastric mucosa (GM) requires a high regeneration rate of epithelial cells during the whole life span. The health of the gastric epithelium highly depends on redox homeostasis, antioxidant defense, and activity of detoxifying systems within the cells, as well as robustness of blood supply. Bioactive products of lipid peroxidation, in particular, second messengers of free radicals, the bellwether of which is 4-hydroxynonenal (HNE), are important mediators in physiological adaptive reactions and signaling, but they are also thought to be implicated in the pathogenesis of numerous gastric diseases. Molecular mechanisms and consequences of increased production of HNE, and its protein adducts, in response to stressors during acute and chronic gastric injury, are well studied. However, several important issues related to the role of HNE in gastric carcinogenesis, tumor growth and progression, the condition of GM after eradication of *Helicobacter pylori*, or the relevance of antioxidants for HNE-related redox homeostasis in GM, still need more studies and new comprehensive approaches. In this regard, preclinical studies and clinical intervention trials are required, which should also include the use of state-of-the-art analytical techniques, such as HNE determination by immunohistochemistry and enzyme-linked immunosorbent assay (ELISA), as well as modern mass-spectroscopy methods.

## 1. Introduction

The gastrointestinal tract (GIT) represents a highly specialized interface between the environment and an organism’s internal medium, aimed primarily to digest food, and absorb nutrients and water. In addition, it fulfils a wide variety of other functions, including, but not limited to, immune defense, excretion of metabolic waste/detoxification, secretory and regulatory functions, and as a physical barrier. Last but not least, it is a vital niche for gut bacteria [1]. The GIT has to withstand harsh conditions, due to exposure to food/chyme, digestive enzymes, different, often very aggressive pH conditions, and numerous bacteria; therefore, high efficiency of protection and regeneration is required for its maintenance and function. This is particularly important in the case of the stomach, whose lumen contains aggressive hydrochloric acid, often reaching pH values of 1–2, and proteolytic enzymes, such as pepsin [2]. Toxins, which may be ingested together with food, as well as some drugs, may contribute to damaging the gastric mucosa (GM). Furthermore, in more than half of the human population worldwide, *Helicobacter pylori* (*H. pylori*) bacteria [3] persist in the GM, and may cause chronic gastritis and peptic ulcer, thus being a major contributor to the pathogenesis of gastric adenocarcinoma and mucosa-associated lymphoid tissue (MALT)-lymphoma [4].

The redox balance is a major homeostatic parameter and a regulatory factor for the metabolic functions of the whole organism and also the GIT [5]. Redox imbalance, often referred to as “oxidative stress”, may be caused either by excessive exposure to oxidants, or by decreased activity of counter-regulatory enzymatic systems and a lack of antioxidants [6]. A certain degree of lipid peroxidation may take place in many cellular processes under physiological conditions, but redox imbalance that is observed in many diseases very often leads to excessive accumulation of oxidized lipids and their degradation products. Among such products of lipid peroxidation, 4-hydroxy-2-nonenal (HNE) is ubiquitous, and one of the most studied compounds, also considered as a “second messenger of free radicals” [7]. HNE is generated from omega-6 fatty acids. Along with its role in the pathogenesis of multiple diseases, it has been shown to be involved in various signaling pathways. It contributes to the regulation of energy metabolism, detoxification, cell proliferation and differentiation, maintenance of the cytoskeleton, and metabolic adaptations to redox derangements [8,9,10].

Considering the sophisticated functions of the mucous membrane and the wide variety of damaging exposures, the maintenance of the redox balance in GM is particularly challenging [5]. In order to sustain the lifelong function of the GIT, the cells of the mucosal epithelium have a high rate of proliferation, and an exceptional regenerative potential. However, this system is prone to derangements, which can result in gastritis, peptic ulcer, and gastric cancer. Gastrointestinal diseases cause severe health problems and overall socioeconomic damage [3]. Progress in understanding the roles of lipid peroxidation and its reaction product, HNE, in health and disease, stimulated studies focused on specific diseases of the GIT. This review is aimed at addressing important issues related to the role of HNE in normal functioning and in the development of diseases of the stomach.

## 2. Approaches to Determine HNE in Samples of Patients Suffering from Stomach Diseases

Along with conventional approaches to measure the concentrations of substances of interest in biological liquids like blood (serum, plasma, and whole blood), urine, cerebrospinal fluid, etc., with several other options available in the case of stomach diseases. First, the stomach is accessible to endoscopy, which is a routine clinical intervention. During endoscopy, it is possible to obtain biopsies of the mucous membrane from different parts of stomach for further morphological studies. Second, gastric juice can be obtained for chemical analysis. Third, a number of “breath-tests” (determination of metabolites of ingested reagents in exhaled air) are available for gastroenterological diagnostics. Finally, feces samples can be taken, for example, to test *H. pylori* bacterial contamination [3]. The researchers have to keep in mind that blood flows from stomach through the portal vein to the liver; many substances, such as xenobiotics, lipid peroxidation products, some hormones, and cytokines, may be degraded there and, thus, may be measured in the peripheral blood within normal concentration ranges, despite evidence of toxicity/inflammation [11].

HNE and other lipid peroxidation products, including acrolein, malonic dialdehyde, and many others, can be measured as biomarkers of redox imbalance [7]. However, their high reactivity and capacity for interactions with multiple functional groups of macromolecules, as well as their transfer to blood and/or urine from other compartments of the organism, may significantly lower steady-state concentrations of free lipid peroxidation products. Most of the detectable HNE are found to be conjugated to proteins or glutathione (GSH). Through a Michael-type reaction of nucleophilic addition, HNE binds covalently to cysteine, lysine, and histidine residues within proteins [12]. Development of specific antibodies against HNE–histidine adducts facilitated further research and enabled implementation of respective analytical methods [13,14].

In this regard, HNE-immunohistochemistry (qualitative/semiquantitative evaluation) is a widely used method of HNE determination, in order to map tissue or intracellular distribution of respective HNE-conjugates in human samples obtained by gastric biopsy [12]. A variety of HNE-ELISAs have been introduced that are applicable for quantitative evaluation of the levels of HNE-adducts in biological fluids, like blood serum, urine, or gastric juice [15]. Other antibody-based methods, which are often applied successfully, include immunofluorescence, immunogold electron microscopy, and immunoblotting [16]. However, since the use of antibodies for analytical purposes is often associated with technical problems, such as inaccessibility of some epitopes and/or their alterations, this may result in incomplete quantification [15]. Furthermore, higher degrees of protein modification can decrease the epitope recognition (non-linear dependence); therefore, the results of analyses based on antibody-dependent techniques should be interpreted very carefully. For clinical purposes, in particular, when histological samples are evaluated, the use of semiquantitative methods for HNE detection may be particularly reasonable [17,18,19,20]. Free HNE can be accurately determined by high performance liquid chromatography, and a number of modifications of mass spectroscopy-based methods [21]. However, due to the high reactivity of free HNE and its low steady-state levels, the determination of HNE conjugates reveals more biologically/clinically relevant information, and may have substantial advantages [12]. The formation of protein conjugates is proportional to the mean levels of free HNE; therefore, antibody-based methods of staining and quantitative determination of HNE are considered to be quite accurate and reliable, especially if HNE–histidine adducts are monitored [12]. The last generation mass spectrometric techniques and instrumentations, in combination with enrichment and separation techniques, have been successfully applied to the determination not only of HNE, but also of its adducts with amino acids in proteins [22,23,24].

## 3. HNE in the Stomach under Physiological Conditions

**Figure 1:**
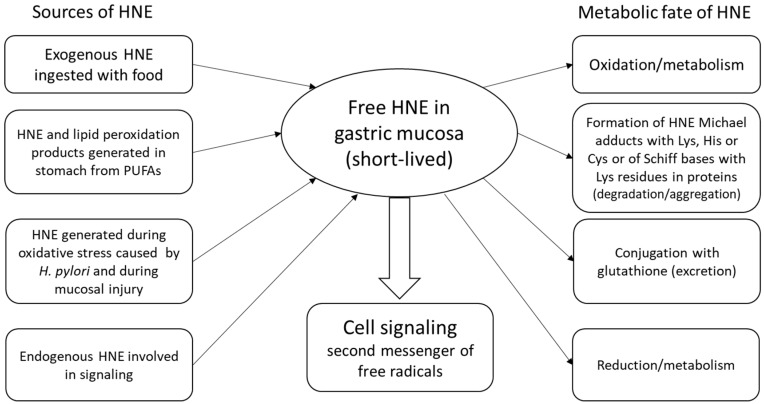
Schematic presentation of major sources of 4-hydroxynonenal (HNE) in gastric mucosa and the ways of its further transformations. Free HNE is a highly reactive molecule, capable of reacting with numerous targets within cells. HNE interfering with redox-sensitive pathways (for example, by binding to cysteine residues) may affect the function of redox-sensitive proteins. Conjugation of HNE with histidine or lysine residues of peptides and proteins are thought to be less important for signaling. However, even in these cases, HNE may bind enzymes, cytokines, and receptors, so they may have important regulatory roles. Hence, such aldehyde-protein adducts can represent a source of HNE and cause secondary oxidative stress, while they can also be used as biomarkers for immunochemical detection of HNE, denoted as advanced lipoxidation end products (ALEs).

In the lumen of the stomach, ingested food is exposed to low pH (hydrochloric acid) and proteolytic enzymes, such as pepsin, contributing to denaturation and degradation of proteins. However, a highly acidic medium facilitates, also, a variety of chemical reactions between different food components [1,2]. Modeling of chemical processes taking place during gastric digestion reveals the possibility of iron- or metmyoglobin-catalyzed generation of substantial amounts of hydroperoxides and other lipid peroxidation products from components of common diets containing meat and unsaturated fats at low pH in the presence of water-dissolved oxygen. Notably, the ingestion of food rich in polyphenols dramatically lowers the generation of hydroperoxides, which may be, at least in part, responsible for the preventive effects of fruits and vegetables [2]. On the other hand, accumulation of lipid peroxidation products in the GM may be enhanced by consumption of large amounts of unsaturated fats that may be a part of many “healthy” diets or popular supplements containing polyunsaturated fatty acids (PUFAs) [25]. Therefore, food products containing significant quantities of PUFAs should be carefully processed and properly stored, in order to prevent their oxidation. Steady-state levels of HNE in the GM result from the rates of their generation/absorption and utilization [26]. The acidity of the chyme may also influence the stability of hydroperoxides and the likelihood of Michael addition within the gastric lumen, whereas the cells of the gastric epithelium are well protected from the acidic content by mucus. Noteworthy, *H. pylori* bacteria produce ammonia to provide protection from hydrochloric acid [27,28] and, therefore, create an alkaline local microenvironment at infection sites, that is more favorable for Michael reactions (Figure 1).

**Figure 2:**
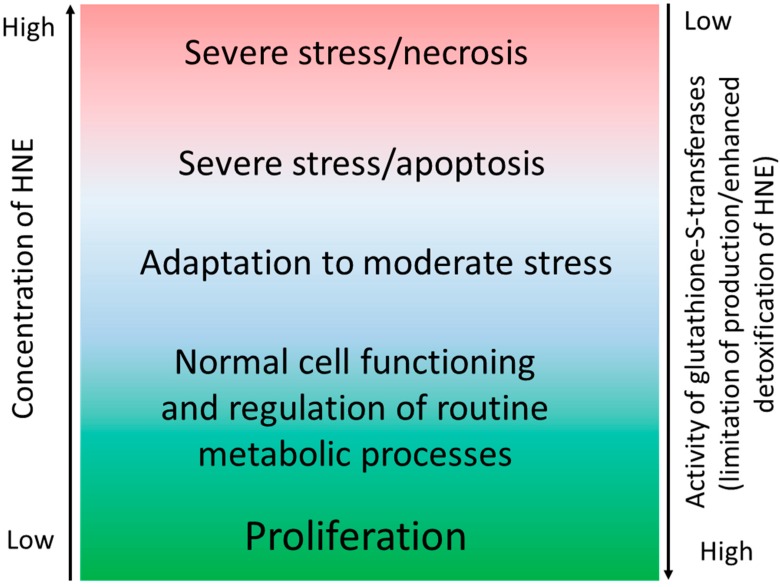
Physiological and pathophysiological effects of HNE on the gastric mucosa depend on the HNE concentration. Steady-state HNE levels inversely correlate with the cellular redox status, and are a function of the rate of its generation and metabolization. HNE content is regulated by the activities of alcohol and aldehyde dehydrogenases, and of glutathione *S*-transferases, depending mostly on the level of reduced glutathione and affinity to cellular proteins [56]. The overall pathophysiological consequences of HNE generation reflect the tissue/cellular redox (im)balance, and depend on the type of cells and the reaction of neighboring cells to the onset of lipid peroxidation. The cells often behave as individuals, not as a homogenous population, which is relevant for carcinogenic effects of HNE and for its involvement in (regulation of) host defense against cancer [57,58,59,60].

Formation of HNE conjugates with glutathione and adducts with proteins may have heterogeneous consequences for the cells, depending on the role of respective residues. Depletion of reduced glutathione may increase vulnerability of the cells to oxidants and shift the redox balance to the pro-oxidant side. Addition of HNE to cysteine residues may alter function of proteins, and may have significant regulatory consequences, whereas binding to other sites (for example, histidine or lysine residues) may have not much effect on function, and can rather reflect the degree of HNE accumulation and possible oxidative damage [12,29,30] (Figure 2).

The epithelium of the GIT is highly proliferating and, depending on the location, it is completely renewed every 3–10 days. Therefore, the immunohistochemical pattern of HNE adducts mainly reflects the metabolic conditions within the mucosa (e.g., oxidative stress, exposure to xenobiotics) during the last few days before taking the sample. Different HNE levels may occur rather as a result of recent alterations, than due to accumulation (for example with age), and are likely to depend on both the renewal rate of epithelial cells and the rate of lipid peroxidation.

A certain degree of accumulation of HNE–histidine adducts in the mucosa of gastric corpus and antrum was demonstrated for the majority of healthy volunteers [19]. Notably, almost all the samples, obtained from asymptomatic apparently healthy subjects, regardless of whether the patients have been *H. pylori*-positive or not, have shown mild to moderate HNE-immunopositivity in the cytoplasm of the gastric glandular epithelium, with only a few HNE-negative samples [19]. A likely explanation of these findings suggests that HNE may play a role in normal signaling and regulation of cellular functions in the GM under physiological conditions. HNE levels appear to be strictly maintained within a homeostatic range, providing adaptations to adverse factors, like metabolic or emotional stress, exogenous toxins that are occasionally ingested with food, or latent *H. pylori* infection. Only excessive and/or prolonged oxidative stress may cause GM injury and inflammation, as discussed below (Figure 2).

Interestingly, most of the *H. pylori*-positive subjects never experience clinically overt forms of gastritis, peptic ulcer, or gastric cancer [31]. This observation is in line with observations that apparently healthy *H. pylori*-positive subjects show no difference in HNE–histidine conjugates in GM compared to controls, despite occasional presence of inflammatory cells in the samples [19]. It is likely that asymptomatic subjects have sufficient compensatory power to cope with the negative influence of the pathogen. Only excessive virulence of certain *H. pylori* strains or lowered resistance of the host may result in clinically significant manifestations. In this regard, it is known that sedentary lifestyle may cause deleterious metabolic changes associated with activation of sympathetic tone (with subsequent parasympathetic impairment) [32]. Genetic defects, psychoemotional stress, and a number of other factors, may also contribute to autonomic imbalance that may lead to increased vulnerability of the GM [33,34,35]. It has been reported that patients with chronic peptic ulcer disease show altered autonomic function, as measured by Holter electrocardiogram monitoring [36]. The relationships of heart rate variability alterations with endothelial dysfunction [37], as well as oxidative stress [38], were earlier noticed. Hence, not clearly intuitive, the relationships of autonomic function and redox balance attract growing attention. For example, an anti-inflammatory action of cholinergic (parasympathetic) signaling [39,40], adrenergic pathways’ interference with H_2_O_2_-mediated insulin signaling [41] and thermogenesis in adipose tissue [42], were demonstrated. Moreover, the link between redox balance and autonomic function was hypothesized [43], and is further confirmed by a recent observation that selective Nrf2 deletion in the rostral ventrolateral medulla in mice evokes hypertension and “sympatho-excitation” [44].

Numerous epidemiological observations associate *H. pylori*-positivity with so called extra-gastric manifestations that include, but are not limited to, atherosclerosis, insulin resistance/diabetes type 2, diseases of liver and pancreas, and others [45,46,47,48,49]. Proposed pathogenesis mechanisms include initial damage of GM caused by *H. pylori* and its virulence factors, oxidative stress and lipid peroxidation, local inflammation, release of pro-inflammatory cytokines and other bioactive mediators to the blood circulation, causing systemic effects and metabolic derangements [4,50,51,52]. Indeed, in *H. pylori*-positive healthy male subjects with a sedentary lifestyle, higher levels of fasting insulin and elevated homeostatic model assessment index (HOMA-index) were observed, compared to *H. pylori*-negative matches [47]. Another study showed significantly increased heart rate and sympathetic tone in *H. pylori*-positive asymptomatic volunteers. However, levels of the water-soluble HNE derivative 1,4-dihydroxynonane mercapturic acid (DHN-MA), iso-PGF2, pro- and anti-inflammatory cytokines, C-reactive protein, and a number of selected hormones, were not different between the groups, indicating that either the degree of local mucosal damage was not strong enough to cause marked elevation of studied parameters, or their mild/moderate elevation is obscured by the passage of blood through the liver [52].

## 4. HNE in Patients with *H. pylori*-Associated Gastritis and Peptic Ulcer

Despite its recent decline, the prevalence of *H. pylori* infection is still very high worldwide, ranging from rates between 20% and 40% in Western countries, to over 90% in many developing countries [3]. There is clear evidence that this microorganism is a causative factor for chronic gastritis type B and peptic ulcer. However, as mentioned above, most *H. pylori*-positive subjects are clinically healthy, and never develop gastritis or ulcer, suggesting that besides *H. pylori* and its virulence factors, conditions of the host organism play a crucial role in the outcome of this complex host–microbe interaction [4,53]. This idea fits well into the framework of the classical concept of balance of factors of “aggression” and “cytoprotection” in GM. On the cellular level, this paradigm is consistent with our current understanding of the principles of redox balance maintenance under stress conditions [6]. GM injury and subsequent inflammation may take place when the capacity of antioxidant mechanisms is not sufficient to protect the cells from the damaging factors and related oxidative stress [5].

Peptic ulcer and gastritis are, for a long time, known to be associated with redox imbalance and excessive lipid peroxidation [54], as confirmed in numerous studies and with different study models [55]. Clinical studies are less abundant, and only a few of them address the issue of oxidative stress and lipid peroxidation in GM. The use of gastric endoscopy enables obtaining of mucosal tissue samples for further histological examination. In the group of *H. pylori*-positive peptic ulcer patients, significantly higher accumulation of HNE–histidine adducts in GM compared to control group was clearly demonstrated [19]. In some cases, severe immunopositivity of nuclei and perinuclear spaces, along with diffuse accumulation of HNE–histidine conjugates in cytoplasm of the cells was observed, pointing to an impaired redox balance in the GM of these patients [18,19].

The pharmacological approach to treat chronic gastritis and peptic ulcer via eradication of *H. pylori* proved to be very successful from the clinical point of view, as it allows most of the patients to be cured of these diseases [61]. In addition, there are reasons to expect that eradication of this microorganism may be useful for prevention and/or treatment of other diseases associated with *H. pylori*, including metabolic syndrome, type 2 diabetes, non-alcoholic fatty liver disease, or atherosclerosis [62,63,64]. How an infection with *H. pylori* may result in systemic pathological effects, as well as the biochemical mechanisms that may contribute to metabolic deteriorations in *H. pylori*-positive patients, needs to be further elucidated.

Despite obvious clinical efficiency, there are reports indicating persistence of HNE–histidine adducts, hyperaccumulation in peptic ulcer patients, even after successful eradication of *H. pylori*, at least in the period of 4 weeks after completing antimicrobial treatment [18]. This is consistent with clinical observations that some patients still have symptoms (epigastric pain, nausea, reduced appetite, etc.) for several months after treatment [65]. It might be possible that metabolic dysfunction in these patients, as an integral part of ulcer disease, contributes to pathogenesis of gastric injury independent of persisting *H. pylori* occurrence. The combination of these two as well as any additional factors is known to increase the risk of ulcerations. In this regard, smoking, psychoemotional stress, unhealthy lifestyle, and suboptimal nutrition may be crucial for the outcome of host–microbial interaction [34,66]. Thus, it depends on the power of intrinsic cytoprotective mechanisms (genetics, sufficient blood microcirculation in stomach, effective autonomic regulation) and exogenous factors (*H. pylori*, ingestion of toxins, and products of PUFA peroxidation), and may vary from long-term asymptomatic carrying to chronic gastritis type B, with the periods of exacerbation and remission, peptic stomach ulcers, and/or duodenum or transformations in the form of MALT-lymphoma or gastric adenocarcinoma.

## 5. HNE in Gastric Carcinogenesis

The GM is exposed to different types of exogenous chemical agents, and reactive species are generated in the stomach during digestion. Some of them may be toxic and cause damage to the gastric epithelium, and some may also be carcinogenic [2]. Chronic inflammation and oxidative stress caused by *H. pylori* infection are also major contributors to malignant transformation of the cells of GM [50,67]. The idea to eradicate *H. pylori* in all carriers, even in asymptomatic ones, is gaining popularity, as some recently published trials showed positive results [68]. Moreover, eradication of *H. pylori* seems to be reasonable also in patients with early stages of gastric cancer undergoing endoscopic resection, since it decreases the rates of metachronous cancers compared to control group [69]. In this context, genotoxicity of supraphysiological levels of HNE and other lipid peroxidation products may be important for carcinogenesis, as well [70,71].

The role of HNE in malignant transformation and growth is ambiguous. On the one hand, HNE can diffuse from the site of generation into the nucleus and bind, covalently, to DNA molecules, causing mutations and supporting carcinogenesis [71], while, on the other hand, it is influencing pathways regulating proliferation, differentiation, and apoptosis of transformed cells. Depending on the activity of detoxifying systems in cancer cells, HNE may be toxic to them or can stimulate their growth and enforce resistance to cytostatic drugs [72].

While, in the case of acute and chronic GM injury caused by *H. pylori* and gastrotoxic agents, oxidative stress and increased lipid peroxidation is well documented, in case of gastric cancer, it is not. As it was shown by Ma et al. (2013), serum levels of major lipid peroxidation products, such as HNE, malonic dialdehyde, conjugated dienes, and 8-iso-prostaglandin F2α, were all decreased in cancer patients compared to control group [73]. Hence, though not statistically significant, lower levels of HNE were also observed in *H. pylori*-positive vs. *H. pylori*-negative patients, that may support the idea that moderate (or local) activation of lipid peroxidation may stimulate systemic activation of detoxification mechanisms through, for example, Nrf2-dependent mechanisms [72].

## 6. HNE in Alcohol- and Non-Steroid Anti-Inflammatory Drug (NSAID)-Induced Gastropathy

Alcohol and a rapidly growing use of NSAIDs are, jointly, the second most important cause of gastric injury after *H. pylori* [66]. Evidence from well-established animal models of GM injury suggests two principal mechanisms responsible for tissue damage. The first, a direct toxic effect on GM, and the second, limitation of gastric microcirculatory blood flow that is essential for a proper rate of proliferation, mucus secretion, etc., through decreased levels of gastroprotective prostaglandin E_2_ with subsequent endothelial dysfunction and autonomic dysregulation, that may cause oxidative stress [74,75]. Both mechanisms contribute to the development of severe local oxidative stress, excessive lipid peroxidation, and accumulation of its products, including HNE, mostly covalently bound to proteins [54].

The important role of autonomic dysregulation is often ignored in case of diseases of stomach. It is known that an elevated sympathetic tone limits blood flow in the organs of gastrointestinal tract, and caused endothelial dysfunction, which is crucial for gastroprotection; therefore, autonomic imbalance may significantly potentiate the damaging effects of alcohol and NSAIDs [76,77].

## 7. Pharmacological and Non-Pharmacological Approaches to Reduce Redox Imbalance in GM

Considering multiple etiologic and pathogenic factors that may interact with each other and contribute to GM damage, there are a number of different approaches in order to prevent or treat gastric injuries (Table 1).

Eradication of *H. pylori* with a combination of two antibiotics and proton pump inhibitors has been proven to be effective in most of the *H. pylori*-positive patients suffering from gastritis and peptic ulcer [61]. However, in some of these patients, elimination of the microbial factor is not sufficient, and symptoms, as well as redox imbalance, may persist long after completion of the treatment [18,65]. Moreover, eradication of *H. pylori* does not significantly lower the risk of gastric cancer, at least within a few years after eradication, and the statistical difference becomes significant only after 8–10 years [78]. Therefore, other approaches are also needed in order to overcome these limitations and to address other aspects of GM injury pathogenesis.

Since substantial amounts of gastrotoxic substances may be ingested with food or generated during digestion, the idea to use drugs, supplements, or certain types of food able to neutralize toxins or reduce the rate of lipid peroxidation was actively explored. Indeed, subjects consuming more fruits and vegetables show lower incidence of gastric diseases, especially gastric cancer [86]. Studies also show that polyphenols reduce the formation of hydroperoxides in stomach and in in vitro models of gastric digestion [2,79]. Pre- and probiotics [87], as well as a number of plant-derived traditional, medicines or extracts, were also shown to be protective against gastric and intestinal mucosal damage and may improve redox balance in mucous membranes in different parts of the GIT [20]. Thus, a number of natural compounds present in fruit and vegetables (e.g., phenolic flavonoids, lycopenes, carotenoids, glucosinolates) act as radical-trapping antioxidants, and they represent not a only useful and convenient beneficial health-promoting approach, due to their natural occurrence and abundance, but also a model for the development of novel drugs aimed to modulate redox balance [88].

The molecular mechanisms underlying protective effects of beneficial compounds are often not yet elucidated, but at least some of them may act via a hormetic response, when moderate prooxidant action causes the activation of defense mechanisms (for example, by induction of target genes of the Nrf-2 transcription factor) [72]. Alternatively, they may contribute to increased mucosal microcirculation through improvement of endothelial function or parasympathetic tone, as it has been shown for Actovegin, which has been used as an anti-ulcer drug for several decades [85]. Among non-pharmacological interventions that showed some efficiency in the case of peptic ulcer disease is also interval hypoxic training [81]. Exact gastroprotective mechanisms in this case are not clear as well, but it is likely that the mechanism includes improvements of autonomic balance and enhanced microcirculation [82].

Therapeutic use of NSAIDs is overwhelming, and in order to reduce their gastrotoxicity, a wide range of new formulations are introduced or are under development [89]. For example, a number of nitric oxide (NO)-, carbon monoxide (CO)-, or hydrogen sulfide (H_2_S)-releasing derivatives of acetylsalicylic acid and other NSAIDs were shown to be as pharmacologically effective as traditional drugs, but have preventive effects against NSAID-induced gastrotoxicity via improvement of endothelial function, and anti-inflammatory and cytoprotective effects [75,76]. Protective actions of these drugs may be also closely related to HNE signaling pathways and maintenance of redox balance in GM.

## 8. Conclusions

The integrity, high functional activity, and sufficient regeneration rate of GM in harsh conditions is very challenging. The health of gastric epithelium highly depends on the efficiency of redox balance maintenance, antioxidant defense, and activity of detoxifying systems within the cells, as well as robustness of blood supply. The products of lipid peroxidation, in particular, of HNE and its protein/histidine adducts, are important mediators in physiological adaptive reactions, cell signaling, and are also implicated in pathogenesis of numerous gastric diseases. Hence, while the mechanisms and consequences of HNE generation in response to strong stressors during acute and chronic gastric injury are well studied, many other important issues related to gastric carcinogenesis, tumor growth and progression, the condition of GM after eradication of *H. pylori*, and many others, still need extensive studies and new comprehensive approaches.

## Figures and Tables

**Table 1:** Selected pharmacological and non-pharmacological interventions and their effects on HNE production/utilization in gastric mucosa.

Intervention	Target Process/Pharmacological Effect	References
Proton pump inhibitors, H_2_ histamine receptor inhibitors	Reduction of acidity, decreased proteolytic activity of gastric juice/decreased gastric injury (production of HNE)	[61,66]
Antibiotics	*H. pylori* eradication/decreased gastric injury (production of HNE)	[61,66]
NO, CO, H_2_S-releasing NSAIDs	Release of CO, NO, and/or H_2_S modulates redox signaling, improves endothelial function, and improves microcirculation/reduced production and improved utilization of HNE	[75,76]
Antioxidants/polyphenols present in food	Reduced lipid peroxidation of PUFAs in stomach/reduced absorption of exogenous HNE	[2,79]
Phytochemical and phytotoxins with moderate prooxidant action	Nrf-2 activators induce expression of antioxidant genes and increase detoxification of HNE	[20,80]
Interval hypoxic training	Improvement of autonomic control of microcirculation and function of internal organs	[81,82]
Exercise, intermittent fasting, caloric restriction	Activation of autophagy, reduction of systemic inflammatory response, improvement of protein quality control and autonomic regulation	[83]
Ulcer-healing drugs (actovegin, solcoseryl etc.)	Mechanism unknown, suggested influence on microcirculation and/or endothelial function	[84,85]
